# Editorial: Biocatalysis and biotransformation guided by protein engineering

**DOI:** 10.3389/fbioe.2023.1159555

**Published:** 2023-02-16

**Authors:** Chao Li, Keke Yang, Heyue Li, Minze Jia, Lijun Guan, Hui-Min Qin

**Affiliations:** ^1^ Key Laboratory of Industrial Fermentation Microbiology of the Ministry of Education, Tianjin Key Laboratory of Industrial Microbiology, College of Biotechnology, National Engineering Laboratory for Industrial Enzymes, Tianjin University of Science and Technology, Tianjin, China; ^2^ Beijing Chengzhi Life Science Co., Ltd., Beijing, China; ^3^ Institute of Food Processing, Heilongjiang Academy of Agricultural Sciences, Harbin, China

**Keywords:** biocatalysts, enzymatic properties, enzyme immobilization, structural analysis, protein design and engineering

Enzyme-based processing technologies have attracted much attention for their wide-ranging applications in the sustainable synthesis of industrially useful products in various fields, including pharmaceuticals, food, feed, chemicals, detergents, and biofuels. Enzymes have significant industrial potential as biocatalysts which display high levels of activity and stereocontrol, function under mild conditions, and can, in principle, be readily modified for industrial uses *via* genetic engineering. Additionally, enzymes have demonstrated their enormous potential for use in green technology as promising alternatives to traditional inorganic catalysts due to the benefits of being sustainable, clean, highly specific, and energy-efficient. Unfortunately, high-performance enzymes are quite scarce in nature, which has severely limited their use in practical synthetic applications. As a result, it has been emphasized that researching and developing highly active and robust biocatalysts that are specifically designed for the biosynthesis of target products is an essential advance. It is extremely valuable to both scientific and industrial interests. Protein engineering based on rational design and directed evolution has been developed as a potent tool to tailor natural enzymes for industrial applications. The substitution of amino acid residues in the protein sequence would induce the redistribution of the conformational ensemble, which can potentially fine-tune the catalytic performance of the enzyme. Meanwhile, enzyme immobilization, which could improve the catalytic properties, recovery, and reusability of enzymes, was also designed for the manufacture of diverse bio-products in large-scale industrial applications to reduce process costs.

This Research Topic has compiled a broad range of original research and reviews to provide readers with an overview of the most recent approaches to biocatalysis and biotransformation guided by protein engineering. Overall, 11 articles on this Research Topic have been published, including 1 review article and 10 research articles. The published articles are briefly highlighted in the following.

Du et al. offered an extensive summary of the principle and process of protein purification, the recent advances and applications of protein purification technologies in the life and health fields, and their wide-ranging effects, which advance the research of protein structure and function, drug development, and precision medicine and provide fresh perspectives to researchers in related fields. Guan et al. developed a novel tannase (TanALb) with comprehensive biochemical and structural characterization to facilitate the biosynthesis of gallic acid by catalyzing the hydrolysis of ester and depside bonds present in hydrolyzable tannins. A new glycosyltransferase (PgUGT) for the production of rebaudioside D was described by Chen et al. Based on two kinds of structure modeling (homology modeling and deep-learning-based modeling), PgUGT was semi-rationally designed using FireProt to enhance its activity and thermostability. Similarly, Chen et al. attempted to improve the enzymatic properties of glucoamylases by domain shuffling between two different original glucoamylases, in which two novel thermostability chimeric glucoamylases were created. Zhang et al. identified a new xylanase from *H. miurensis* (Hmxyn) with wheat arabinoxylan hydrolysis activity. The water-unextractable arabinoxylan was significantly degraded by the use of recombinant Hmxyn, which also enhanced the dough’s organizational structure, air-holding capability, and expansion rate. Two tandem PETase-like hydrolases (Ples), Ple628 and Ple629, were examined in terms of their kinetic characteristics, degradation products, and activity by Cifuentes et al. Structural analysis revealed that two enzymes were classified as member of the PETases IIa subclass, α/β hydrolase superfamily since their structures were similar to other PETases. Jiang et al. developed a procedure for the type 1 copper site active center cluster model to determine the redox potentials of copper efflux oxidase. The target cluster model structures were designed using the equilibrium structures from the dynamics simulations, whose oxidized and reduced states were geometrically optimized independently in the solvated environment at the B3LYP-D3(BJ)/6-311G* level. Yang et al. improved the reusability of a novel D-allulose-3-epimerase (RpDAE) by *in situ* encapsulation within the microporous zeolite imidazolate framework at room temperature. Wang et al. optimized the conversion of flavonoid glycosides and their analogs to their lipophilic ester derivatives using immobilized *Thermomyces lanuginosus* lipase (TLL) as nanobiocatalysts, in which TLL was immobilized on polydopamine-functionalized magnetic Fe_3_O_4_ nanoparticles. Miao et al. successfully immobilized myoglobin (Mb) using mesoporous silica sieves (SBA-15), which were synthesized with various pore sizes using a hydrothermal process. The immobilized Mb, especially the crosslinked version, showed good reusability and stability. It showed great significance to the industrial application. Additionally, Miao et al. further optimized the protein loading capacity, thermostability, storage stability, and reusability of the SBA-15 by functionalizing the surface of the mesoporous molecular sieve.

We hope this Research Topic will benefit both the scientific and industrial communities to track the state-of-the-art in this enzymatic field, no matter whether the readers are at a beginner or professional level. Finally, we would also like to express our appreciation to all of the contributing authors for sharing their excellent scientific works, the reviewers for their insightful comments, as well as the editorial staffs of Frontiers in Bioengineering and Biotechnology for their unwavering support to ensure the success of this Research Topic.

